# Mechanism of Abnormal Activation of MEK1 Induced by Dehydroalanine Modification

**DOI:** 10.3390/ijms25137482

**Published:** 2024-07-08

**Authors:** Yue Zhao, Shan-Shan Du, Chao-Yue Zhao, Tian-Long Li, Si-Cheng Tong, Li Zhao

**Affiliations:** 1 School of Life Sciences, Jilin University, Changchun 130118, China; yue_zhao20@mails.jlu.edu.cn (Y.Z.); duss1322@mails.jlu.edu.cn (S.-S.D.); zhaocy1321@mails.jlu.edu.cn (C.-Y.Z.); 2 State Key Laboratory of Supramolecular Structure and Materials, Jilin University, Changchun 130012, China; litl23@mails.jlu.edu.cn (T.-L.L.); tongsc22@mails.jlu.edu.cn (S.-C.T.)

**Keywords:** MEK1, Dha modification, MEK1 inhibitors, molecular dynamics simulations

## Abstract

Mitogen-activated protein kinase kinase 1 (MAPK kinase 1, MEK1) is a key kinase in the mitogen-activated protein kinase (MAPK) signaling pathway. MEK1 mutations have been reported to lead to abnormal activation that is closely related to the malignant growth and spread of various tumors, making it an important target for cancer treatment. Targeting MEK1, four small-molecular drugs have been approved by the FDA, including Trametinib, Cobimetinib, Binimetinib, and Selumetinib. Recently, a study showed that modification with dehydroalanine (Dha) can also lead to abnormal activation of MEK1, which has the potential to promote tumor development. In this study, we used molecular dynamics simulations and metadynamics to explore the mechanism of abnormal activation of MEK1 caused by the Dha modification and predicted the inhibitory effects of four FDA-approved MEK1 inhibitors on the Dha-modified MEK1. The results showed that the mechanism of abnormal activation of MEK1 caused by the Dha modification is due to the movement of the active segment, which opens the active pocket and exposes the catalytic site, leading to sustained abnormal activation of MEK1. Among four FDA-approved inhibitors, only Selumetinib clearly blocks the active site by changing the secondary structure of the active segment from α-helix to disordered loop. Our study will help to explain the mechanism of abnormal activation of MEK1 caused by the Dha modification and provide clues for the development of corresponding inhibitors.

## 1. Introduction

**Figure 1:**
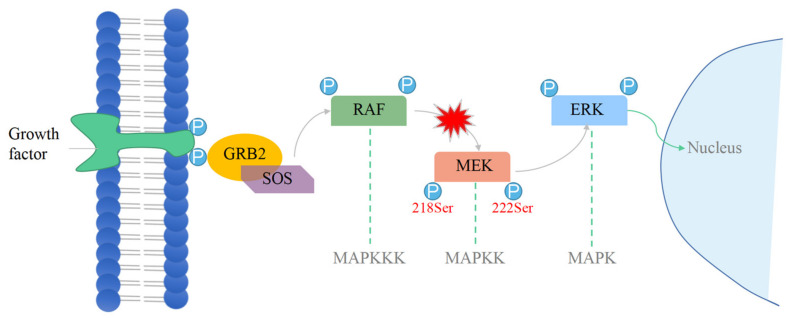
MAPK signaling pathway.

The mitogen-activated protein kinase (MAPK) signaling pathway is one of the most important intracellular signaling pathways, playing a key role in cellular processes, such as proliferation, apoptosis, gene regulation, differentiation, and cell motility [1,2]. It consists of three different protein kinase modules, MAPK, MAPK kinase (MAPKK), and MAPK kinase kinase (MAPKKK), catalyzing a series of phosphorylation events (Figure 1) [3]. MEK1 (mitogen-activated protein kinase kinase 1) belongs to the MAPKK family and is the critical node in the MAPK signaling pathway. In normal physiological conditions, MEK1 is phosphorylated by RAF kinase (a member of the MAPKKK family) [4]. After phosphorylation, the activated fragment refolds to provide the enzyme’s active conformation and further phosphorylates downstream kinase ERK1 (extracellular signal-regulated kinases, a member of the MAPK family), thereby regulating downstream target gene expression and cell function [5].

Abnormal activation and dysfunction of MEK1 have been shown to be associated with a variety of human diseases, such as breast cancer, lung cancer, and melanoma, making it an important target for cancer therapy [6,7,8,9,10,11,12,13,14]. Dozens of abnormal activation forms related to MEK1 have been found in tumor and developmentally abnormal cells, 60% of which involve activating mutations, such as K57N (lung adenocarcinoma) [15], P124S (melanoma) [11], E203K (skin syndrome) [16], I103N (skin syndrome) [17], and F53L (melanoma and skin syndrome) [18]. These mutations disrupt the stability of the inactive conformation and make MEK1 more susceptible to Raf-mediated phosphorylation.

Recently, a novel MEK1 abnormal activation mechanism was discovered, which is the result of dehydroalanine (Dha) modification and has the potential to be carcinogenic [19]. Ser218 and Ser222 can be modified by Dha to maintain MEK1 kinase activity [20]. Previously, it has been reported that Dha modification can be achieved in various ways, such as changes in the extracellular environment and intracellular metabolic pathways [21,22,23,24], which contain virulence factors that can eliminate phosphate from pSer/pThr in the kinase active segment to produce Dha [25]. However, the specific mechanism of this Dha modification in the MEK1 activation process is not yet clear.

Molecular dynamics (MD) simulations are indeed a valuable tool to study protein structural and functional changes, as they can provide atomic-level structural and dynamic information [26,27]. MD simulations have been successfully used to investigate the dynamic processes of protein mutations and post-translational modifications (PTMs) [28]. Lee et al. used molecular dynamics simulations to explore two regulatory states of SnRK2.6 (sucrose nonfermenting-related protein kinase 2.6): persulfidation and phosphorylation. They elucidated how these modifications fine-tune the “on” and “off” states of SnRK2.6, thereby controlling the initiation and braking processes of ABA signal transduction [29]. Zhu et al. clarified the mechanism by which active mutations weaken the inhibitory effect of trametinib on MEK1 by simulating the dissociation pathways of wild-type MEK1 and two active mutants with trametinib [30].

This work aims to explore the mechanism of MEK1 abnormal activation caused by a Dha modification, investigate its role and mechanism in MEK1 abnormal activation, and predict the inhibitory effects of four existing FDA-approved MEK1 inhibitors on Dha modification-induced MEK1 abnormal activation [31,32,33]. The MD simulation method was used to explore and analyze the conformation space of wild-type MEK1 protein (WT_MEK1), phosphorylated MEK1 (the same sequence with wild-type MEK1 but phosphorylated on Ser218 and Ser222, PP_MEK1), and Dha-modified MEK1 (the same sequence with wild-type MEK1 but with dehydroalanine (Dha) modifications on Ser218 and Ser222, Dha_MEK1). It is worth noting that WT_MEK1 is an inactive enzyme, PP_MEK1 is a normal active enzyme, and Dha_MEK1 is an abnormally activated enzyme. To overcome the energy barriers between active and inactive forms of MEK1, we also utilized metadynamics to expand the scope of traditional molecular dynamics simulations [34]. Then, the docking method was used to study the effects of inhibitors on the catalytic activity of Dha_MEK1. The results of this study are expected to shed light on the role of Dha modification in the MEK1 signaling pathway and provide clues for the development of corresponding inhibitors.

## 2. Results and Discussion

### 2.1. Mechanism of MEK1 Aberrant Activation Induced by Dehydroalanine Modification

#### 2.1.1. Dha Modifications Mainly Affect Conformational Change of Active Segment

**Figure 2:**
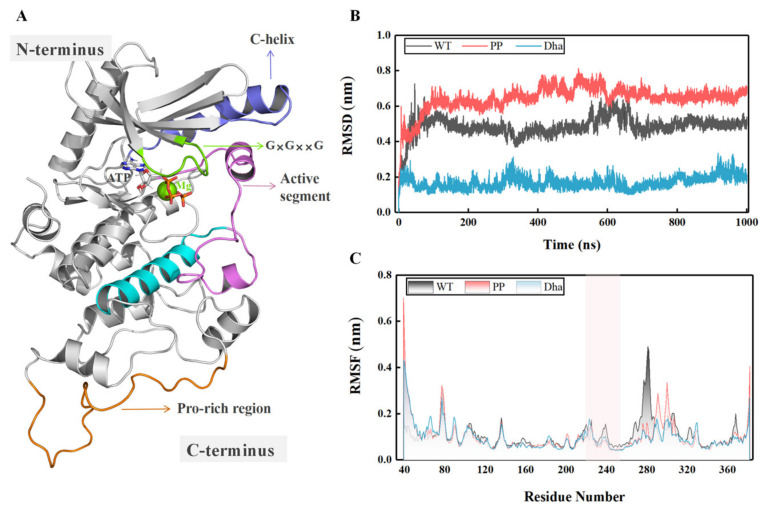
(**A**) Representation of MEK1 and its functional domains. (**B**) Time-dependent changes in the root mean square deviation (RMSD) values during the molecular dynamics (MD) simulations. (**C**) Root mean square fluctuation (RMSF) of the protein backbone after 200 ns of MD simulations.

This study focuses on three systems, namely WT_MEK1, PP_MEK1, and Dha_MEK1. MEK1’s structure and functional domains are shown in Figure 2A. Details about the construction of the systems are described in Section 3. These systems were subjected to 1 μs of MD simulations, and the stability of their structures was analyzed. Firstly, the root mean square deviation (RMSD) of the protein backbone was calculated to evaluate the stability of the MD simulations. As shown in Figure 2B, the RMSD values of the three systems stabilized after 100 ns simulations. The average RMSD values for WT_MEK1, PP_MEK1, and Dha_MEK1 after 200 ns simulations are approximately 0.51 ± 3.83 Å, 0.65 ± 4.07 Å, and 0.22 ± 4.59 Å, respectively, indicating that the structures of the three systems reach relative equilibrium and could be used for subsequent analysis. Next, to analyze the fluctuation in MEK1 residues, the root mean square fluctuation (RMSF) values of the backbone residues in the three systems were calculated. From Figure 2C, it can be observed that the peaks of the MEK1 active segment (Val211-Tyr236, the pink domain in Figure 2A) and the Pro-rich region (Val261-Glu291, the orange domain in Figure 2A) show significant differences among the three systems. Compared to the WT_MEK1 system, the residue fluctuations in the PP_MEK1 and Dha_MEK1 systems are relatively weaker. In conclusion, the modifications mainly affect the fluctuations in the MEK1 residues in the active segment.

**Figure 3:**
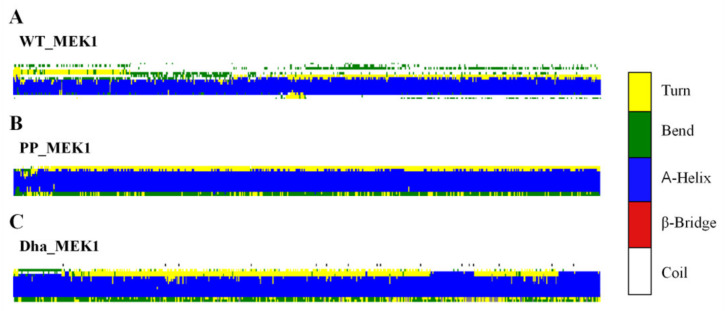
Dynamic changes in the secondary structures of the active segment throughout the entire simulation process, (**A**) WT_MEK1, (**B**) PP MEK1, and (**C**) Dha MEK1.

Next, the secondary structure differences of the active segment in the WT_MEK1, PP_MEK1, and Dha_MEK1 systems during the simulation were analyzed. The results show that the α-helical content of the active segment (Val211-Tyr236) significantly increases in the Dha_MEK1 system, as shown in Figure 3. This suggests that the modification of Dha promotes the stability of the helical structure in the active segment of MEK1, which is consistent with the RMSF results.

**Figure 4:**
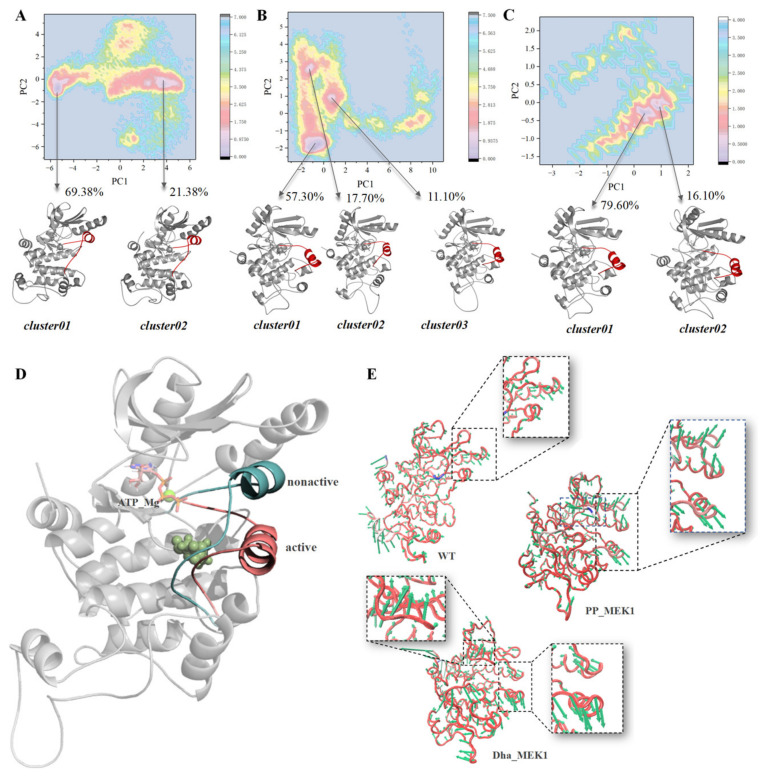
(**A**–**C**) FEL analysis of the WT_MEK1, PP_MEK1 and Dha_MEK1 respectively. The energy-minimized representative conformations are shown, with the active segment highlighted in red and the rest of the conformations colored in gray using a cartoon representation. (**D**) Comparison of representative structures of the WT_MEK1 and Dha_MEK1 systems (blue helix: active segment of WT_MEK1, pink helix: active segment of Dha_MEK1). The catalytic site (Asp190) is depicted as a sphere (light green), and ATP and Mg^2+^ ions are shown as sticks (colored according to the elements). (**E**) Hedgehog plot of MEK1 based on the first principal component (PC) of the WT_MEK1, PP_MEK1, and Dha_MEK1, with arrows indicating the direction and magnitude of motion trends.

The free energy landscape (FEL) is a two-dimensional plot that describes the relative stability of molecular conformations and obtains the representative conformation of a molecule. After conducting principal component analysis (PCA), we extracted representative conformations of the WT_MEK1, PP_MEK1, and Dha_MEK1 systems from the free energy landscape plot (as shown in Figure 4A–C). The results also show that the changes in the WT_MEK1, PP_MEK1, and Dha_MEK1 systems mainly occur in the active segment. In the Dha_MEK1 system, the first cluster (cluster 01) accounts for 79.60% of all conformations, while in the PP_MEK1 system, the first cluster accounts for only 57.30%. Furthermore, the representative conformations of the WT_MEK1 and Dha_MEK1 systems were superimposed (Figure 4D). The conformational comparison reveals that while most regions of the MEK1 kinase structure are conserved, there is a significant movement of the active segment with approximately 10.24 Å towards the C-terminus of the kinase after the Dha modification.

Since the motions of the active segment impact the function of MEK1, we analyzed the protein’s motion trends using the first frame conformation from the trajectory and the average conformation based on RMSD clustering, represented by a porcupine plot (Figure 4E). The hedgehog plot is an effective method for analyzing the direction and range of the main motions in the backbone residues of biomolecular systems. From a whole-protein perspective, the WT_MEK1 system exhibits only slight motion intensity, while the PP_MEK1 and Dha_MEK1 systems show high-intensity motion, especially in the Pro-rich region, the active segment, and C-helix. Additionally, the hedgehog plot of the Dha_MEK1 system clearly shows a more obvious motion trend in its G×G××G domain compared to that of the WT_MEK1 and PP_MEK1 systems.

**Figure 5:**
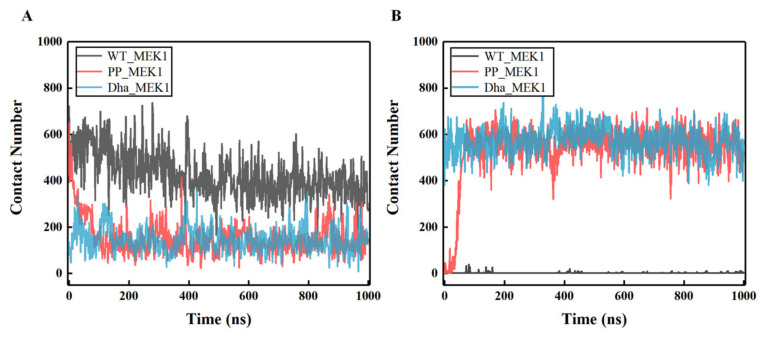
The contact number variation over time between (**A**) the active segment and the N-terminal and (**B**) the active segment and the C-terminal.

To further analyze the motion of the active segment, the contact numbers between the active segment and the N-terminal and between the active segment and the C-terminal were calculated over time using MD analysis (with a radius cutoff 5.0 nm). From the data in Figure 5A, it can be observed that the average contact number between the active segment and the N-terminal of the WT_MEK1 remains around 450, consistently higher than that of the PP_MEK1 and Dha_MEK1 systems. The contact number of PP_MEK1 decreases from around 640 to 190 at about 100 ns, while the contact number of Dha_MEK1 stabilizes at about 170. The results in Figure 5B show the contact number between the active segment and the C-terminal, indicating that the interactions between the active segment and the C-terminal in WT_MEK1 almost disappeared. However, the contact number of PP_MEK1 and Dha_MEK1 is significantly higher than that of WT_MEK1. In summary, weakened interactions between the active segment and the N-terminal and the enhancement in interactions between the active segment and C-terminal induce the activation of MEK1, regardless of normal or abnormal activation.

#### 2.1.2. Characteristics of the Active Pocket

**Figure 6:**
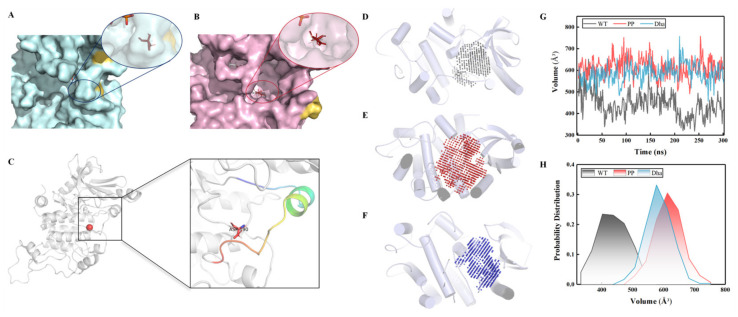
Structure and volume of the active pocket. (**A**) WT_MEK1 and (**B**) Dha_MEK1 active pocket structure. The representative conformation is obtained through conformation clustering and represents the highest probability conformation, confirming the stability of the observed states. (**C**) Representation of the active pocket with key catalytic residue Asp190 highlighted (red sphere). (**D**) WT_MEK1, (**E**) PP_MEK1 and (**F**) Dha_MEK1 catalytic pocket volume. (**G**) Variation in the catalytic pocket volume over time. (**H**) Probability distribution of the catalytic pocket volume.

In the representative conformation of the WT_MEK1, the catalytic pocket remains in a closed state (Figure 6A). However, in the active state of MEK1 (both normal and abnormal activation), the catalytic pocket is fully exposed (Figure 6B), leading to the significant exposure of the catalytic residue Asp190 [35] (Figure 6C). Using the POVME 3.0 software package [36], we calculated the volume of the catalytic pocket cavity using the equilibrium simulation trajectories of the three systems, spanning 700 to 1000 ns. Remarkably, there are significant differences in the volume of the catalytic pocket cavity among the WT_MEK1, PP_MEK1, and Dha_MEK1 systems (Figure 6D,E). It can be clearly observed that the volume range of the cavity in the Dha_MEK1 system is similar to that of the PP_MEK1 system, fluctuating around 600 Å^3^, while the pocket volume of the WT_MEK1 is around 450 Å^3^ (Figure 6G,H). These results suggest that the active conformation of Dha_MEK1 is stable, and its catalytic cavity is persistently open. This feature is similar to normal activation, indicating that both the catalytic pockets in normal and abnormal states are more exposed, which is a characteristic of MEK1 transitioning from an inactive to an active state.

**Figure 7:**
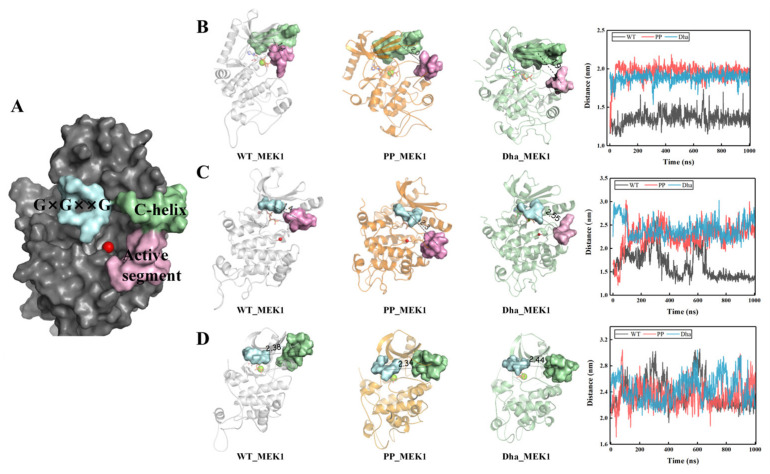
(**A**) Representation of G×G××G domain (light blue), the C-helix (green), and the active segment (pink). (**B**) Visualization of the distance between the C-helix and the active segment and its variation over time. (**C**) Visualization of the distance between the G×G××G domain and the active segment and its variation over time. (**D**) Visualization of the distance between the C-helix and the G×G××G domain and its variation over time.

Further investigation of the MEK1 catalytic pocket revealed that the distance variations between three segments at the entrance of the pocket (G×G××G domain, C-helix, and active segment, shown in Figure 7A) effectively characterize the opening process of the catalytic pocket. Firstly, we calculated the distance between the active segment and the C-helix over time (Figure 7B). The average distances in the three systems are 1.42 nm, 2.30 nm, and 1.94 nm, respectively, consistent with previous studies [37], showing that upon MEK1 activation, the C-helix loses control over the active segment, resulting in an increased distance between them. Secondly, we calculated the distance between the active segment and the G×G××G domain over time (Figure 7C). The average distances in the three systems are 1.45 nm, 2.31 nm, and 2.55 nm, respectively. Finally, we calculated the distance between the G×G××G sequence and the C-helix over time (Figure 7D). The average distances in the three systems are 2.38 nm, 2.34 nm, and 2.44 nm, respectively, indicating that normal and abnormal activation of MEK1 does not significantly affect the interaction between the G×G××G domain and the C-helix. In summary, the relative movements of the three segments at the entrance of the catalytic pocket represent the transition of MEK1 from a closed conformation in the wild type to an open conformation upon activation. This transition is necessary for the activation of MEK1 and its downstream substrate ERK.

#### 2.1.3. Driving Forces behind the Active Segment Conformational Changes

**Figure 8:**
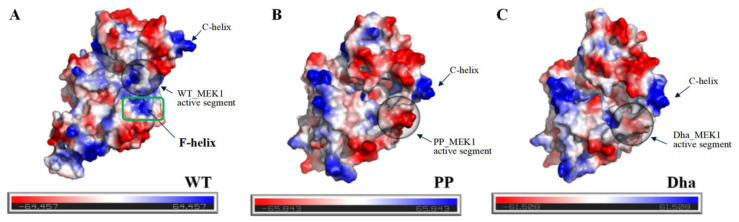
Electrostatic potential surfaces (EPS) colored based on the electrostatic potential of (**A**) WT_MEK1, (**B**) PP_MEK1 and (**C**) Dha_MEK1. In the figures, the positions of Ser218 and Ser222 in the active segment are enclosed in black circles and the F-helix is outlined in green. The red color corresponds to ESP values below −64.457 kcal/e.u, the blue color corresponds to ESP values above +64.457 kcal/e.u, and the gray color corresponds to ESP values between −64.457 and +64.457 kcal/e.u.

We analyzed the protein’s electrostatic potential surfaces using the Adaptive Poisson–Boltzmann Solver (APBS) plugin in PyMOL [38]. Figure 8A–C show the electrostatic potential surfaces of WT_MEK1, PP_MEK1, and Dha_MEK1. In WT_MEK1, the total net charge is neutral (Figure 8A). PP_MEK1 and Dha_MEK1 are negative due to the addition of phosphate and Dha (Figure 8B,C). The additional negative charge causes the active segment to move towards the positive regions (F-helix, the green box in Figure 8A) in PP_MEK1 and Dha_MEK1, resulting in the transition of MEK1 into its active state. Therefore, the positive C-helix moves down in PP_MEK1 and Dha_MEK1. Overall, electrostatic interactions drive the movement of the active segment, crucial for maintaining MEK1 in its active structure.

**Figure 9:**
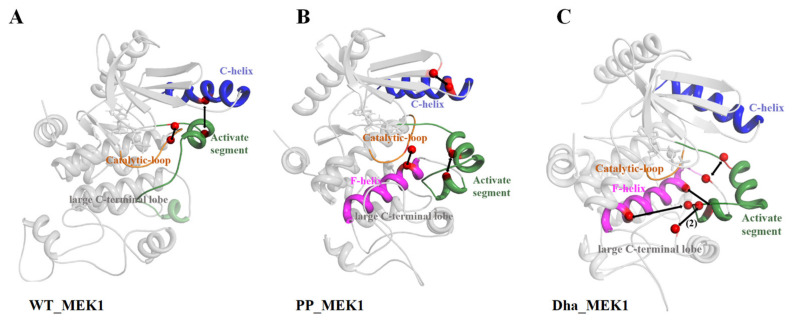
Structural representations of hydrogen bonds. (**A**) WT_MEK1, (**B**) PP_MEK1, and (**C**) Dha_MEK1. MEK1 is depicted in gray cartoon representation, with the C-helix highlighted in blue, the active segment in green, and the F-helix in pink. Amino acid residues are represented by red spheres, and the interactions are indicated by black arrows.

To further understand the interactions driving the active segment movements in the three MEK1 systems, we analyzed the hydrogen bond variations in the WT_MEK1, PP_MEK1, and Dha_MEK1 systems. As shown in Table 1, compared to WT_MEK1, hydrogen bond occupancy in the PP_MEK1 and Dha_MEK1 systems increases significantly, mainly in the activation segment residues (Val211-Tyr236), including Arg108-Asp136, Arg189-Ser241/Asp245, Gly213-Tyr240, Arg234-Asp217, Arg305-Ser228, and Ser244-Glu233. As shown in Figure 9, the new-forming hydrogen bonds are primarily located along the F-helix of MEK1. The hydrogen bond between Arg305 and Ser228 is specifically present in Dha_MEK1. The formation of these hydrogen bonds promotes the open conformation of the active segment.

#### 2.1.4. Metadynamic Simulations Indicate That Dha Modifications Cause MEK1 in a Continuous Activated State

**Figure 10:**
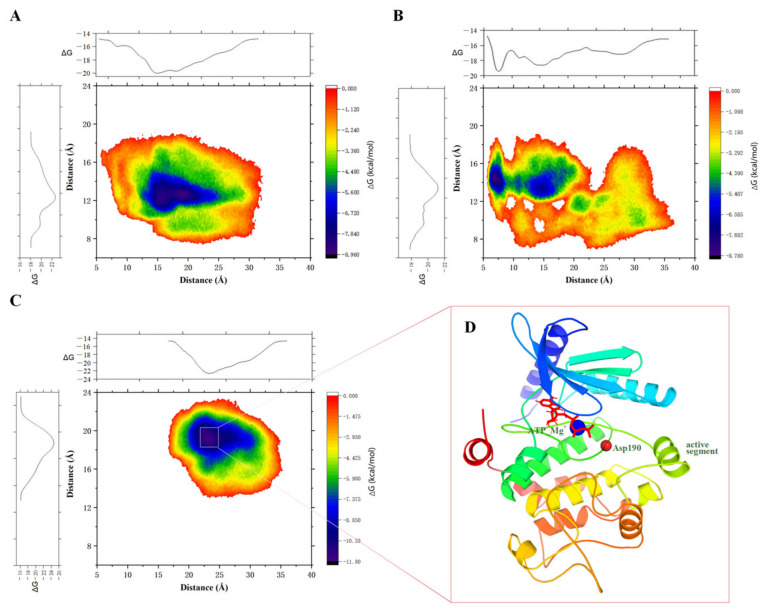
The Free energy landscapes of (**A**) WT_MEK1, (**B**) PP_MEK1 and (**C**) Dha_MEK1, along with the one-dimensional projections of the two collective variables (CVs). (**D**) Representative conformation corresponding to the energy minima in the Dha_MEK1 system.

We employed the Well-Tempered Metadynamic Simulations to further explore the conformational space of WT_MEK1, PP_MEK1, and Dha_MEK1. Based on the analysis ahead, two collective variables (CVs) were defined to characterize the free energy landscape (FEL) of the three systems (Figure 10), the distance between the active segment and the C-helix (CV1), and the distance between the active segment and the G×G××G domain (CV2).

In the WT_MEK1 system, the distances primarily fluctuate around 1.75 nm and 1.30 nm, respectively, for CV1 and CV2, indicating limited exposure of the catalytic cavity (Figure 10A). For the PP_MEK1 system, the FEL exhibits three distinct states, closed, open, and intermediate. Notably, free energy barriers of the transition state and open state are −4.566 kcal/mol and −8.780 kcal/mol, respectively (Figure 10B). This suggests a transition between open and closed states after phosphorylation. In contrast, the Dha_MEK1 system displays a global minimum free energy of −11.80 kcal/mol (Figure 10C). These findings underscore the significance of active segment movement and catalytic pocket exposure in MEK1 activation. Upon Dha modification, MEK1remains in an energy valley with the active conformation, thus remaining in a persistently activated state. Based on the FEL analysis of the Dha_MEK1 system, we derived a stable structure of Dha-modified MEK1; the stable conformation was obtained by clustering the trajectory based on the distance between the activation loop and the C-helix (CV1). The conformation with the highest probability after clustering was identified as the representative conformation of Dha-modified MEK1 (Figure 10D).

**Figure 11:**
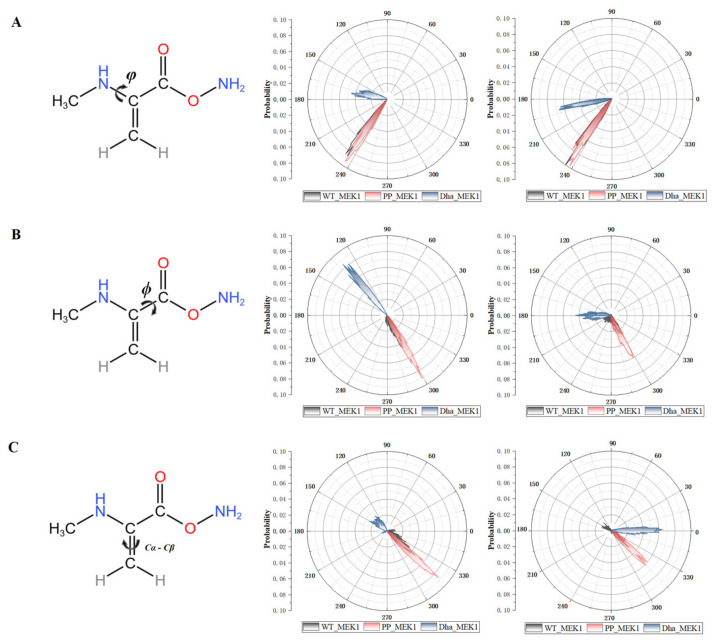
Representation and probability distribution of the dihedral angles (**A**) *φ*, (**B**) *ϕ* and (**C**) Cα-Cβ. Probability distributions shown in unit circle are for residues at positions 218 (left) and 222 (right).

Previous studies mentioned that Dha exhibits limited rotational freedom, leading to the increased planarity of the phi, psi, and Cα-Cβ angles [24,39,40]. We analyzed the time-dependent changes in dihedral angles (phi, psi, and Cα-Cβ) at Dha-modified Ser218 and Ser222 (Figure 11). For the Dha_MEK1 system (blue plot in Figure 11), we observed the following ranges: (1) phi: 120° < φ < 180° and −180° < φ < −150°; (2) psi: 120° < ϕ < 150° and 150° < ϕ < 180°; and (3) Cα-Cβ: 100° < Cα-Cβ < 180° and −30° < Cα-Cβ < 30°. Compared to WT_MEK1 (black plot in Figure 11) and PP_MEK1 (red plot in Figure 11), the Dha structure in the Dha_MEK1 system exhibits significantly increased planarity. In summary, our analysis indicates that Dha modification may affect the conformation of MEK1 by altering protein dihedral angles, thereby enhancing its stability and the stability of the active segment.

### 2.2. Prediction of the Inhibitory Effects of FDA-Approved Drugs on the Catalytic Activity of Dha-Modified MEK1

#### 2.2.1. Stability Analysis of Docking Structures

To predict the blocking effect of FDA-approved MEK1 inhibitors on Dha_MEK1, we docked Trametinib, Cobimetinib, Binimetinib, and Selumetinib on Dha_MEK1. These non-competitive inhibitors were subjected to docking simulations with Dha_MEK1, following by 500 ns molecular dynamics simulations. Inhibitor and Dha_MEK1 complexes were labeled as follows: Dha_MEK1_Trametinib (Dha_MEK1_Tra), Dha_MEK1_Selumetinib (Dha_MEK1_Sel), Dha_MEK1_Cobimetinib (Dha_MEK1_Cob), andDha_MEK1_Binimetinib (Dha_MEK1_Bin).

**Figure 12:**
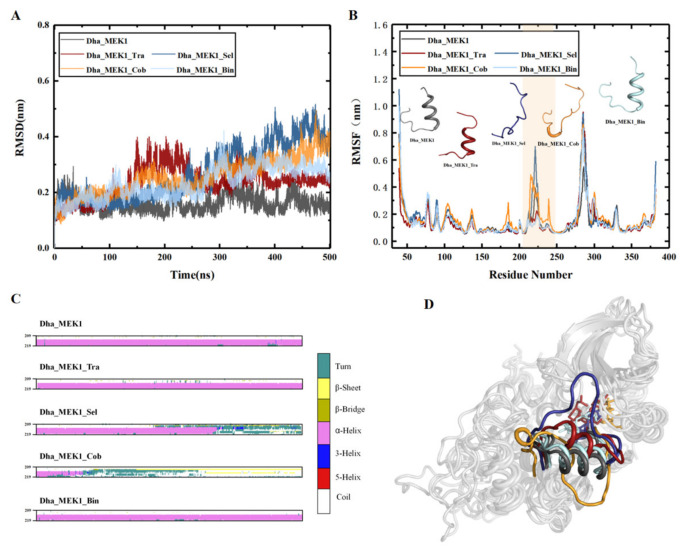
(**A**) RMSD of Dha_MEK1 and Dha_MEK1 with four inhibitor complexes. (**B**) RMSF and the secondary structure of the active segment (Val211-Tyr236). (**C**) Time-dependent changes in the secondary structure of the active segment (Phe209-Met219). (**D**) Representation of clustered conformations, active segment was highlighted in gray, red, dark blue and orange for Dha_MEK1, Dha_MEK1_Tra, Dha_MEK1_Sel, Dha_MEK1_Cob and Dha_MEK1_Bin respectively. The plots were created using LigPlot [41].

RMSDs were calculated to assess the stability of the four complex systems. As depicted in Figure 12A, the RMSD values for Dha_MEK1, Dha_MEK1_Tra, and Dha_MEK1_Bin stabilized after 300 ns, while RMSD values for Dha_MEK1_Sel and Dha_MEK1_Cob increased. The increasing RMSDs indicate larger conformational changes, and such conformational changes are continuous, which will be discussed later. Next, we calculated RMSF, shown in Figure 12B. RMSF values of active segments in the Dha_MEK1_Sel and Dha_MEK1_Cob systems show higher flexibility. Moreover, from the secondary structure data (Figure 12C), we observed that the active segment transitions from a helical structure to an unstructured coil after binding with Selumetinib and Cobimetinib. In contrast, the active segments in the Dha_MEK1, Dha_MEK1_Tra, and Dha_MEK1_Bin systems remain stable with minimal fluctuations and maintain the α-helix secondary structure. The influences of Selumetinib and Cobimetinib on the active segment structure changes are shown in Figure 12D. In the Dha_MEK1_Sel and Dha_MEK1_Cob systems, the active segment’s structure (highlighted in deep blue and orange) deviates from its original position, fluctuating over the active pocket. Conversely, in the Dha_MEK1_Tra and Dha_MEK1_Bin systems (highlighted in red and light blue), there are no significant structural changes, and the active segments maintain a stable conformation similar to that of Dha_MEK1 (highlighted in gray). In summary, binding of Selumetinib and Cobimetinib to Dha_MEK1 causes the stable α-helix in the active segment to transition into an unstructured coil, disrupting the stable activated conformation of the Dha_MEK1 system.

**Figure 13:**
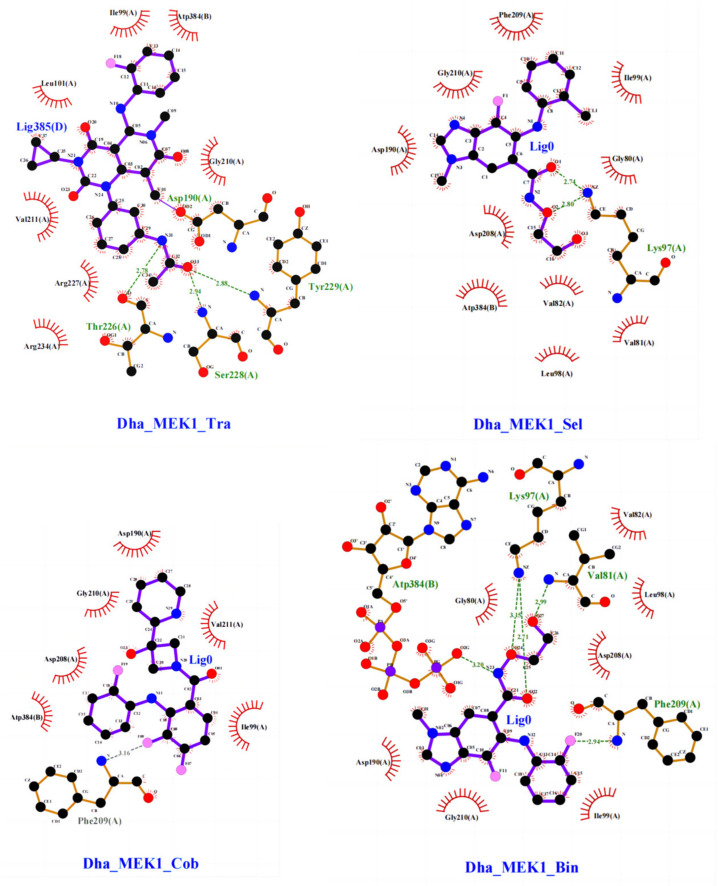
The interactions between Dha_MEK1 and Trametinib, Selumetinib, Cobimetinib and Binimetinib respectively. Residues involved in hydrogen bond and ligand binding are shown as stick models, while non-bonded interactions are depicted as red brush-like structures. Hydrogen bonds are depicted as green dashed lines with corresponding bond lengths in angstroms (Å). Carbon atoms are represented as black spheres, nitrogen atoms as blue spheres, oxygen atoms as red spheres, fluorine atoms as pink spheres and phosphorus atoms as purple spheres.

Interactions between the inhibitors and the protein were analyzed, especially the hydrogen bonds and hydrophobic contacts, as shown in Figure 13. In the Dha_MEK1_Tra system, Trametinib forms hydrogen bonds with Asp190, Tyr226, Ser228, and Tyr229. In the Dha_MEK1_Sel system, Selumetinib forms a hydrogen bond with Lys97. In the Dha_MEK1_Cob system, Cobimetinib forms a hydrogen bond with Phe209. In the Dha_MEK1_Bin system, Binimetinib forms hydrogen bonds with Val81, Lys97, Phe209, and ATP. These interactions contribute to the binding of the inhibitors.

#### 2.2.2. The Inhibitory Effect of Selumetinib on MEK1 Abnormal Activation

**Figure 14:**
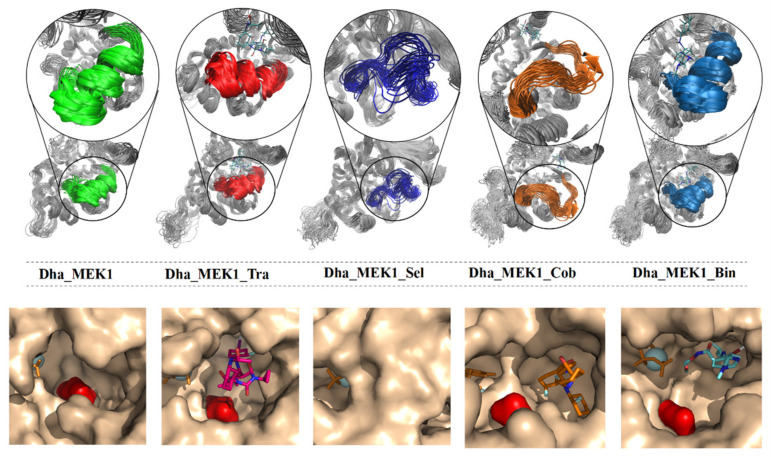
Representation of the conformational distribution of the active segment in the catalytic region binding with Trametinib, Selumetinib, Cobimetinib and Binimetinib respectively. Asp190 is represented in surface colored in red, the ATP is in licorice colored in orange, the Mg^2+^ ions are represented by van der Waals spheres, colored in light blue. The four inhibitors in the active pocket are drawn in licorice, and the atoms different of oxygens are colored in red (Trametinib), orange (Selumetinib and Cobimetinib) and light blue (Binimetinib). The other protein residues are drawn by surface colored in beige.

We observed changes in the active segment due to the binding of Selumetinib and Cobimetinib inhibitors. To investigate their impact on the catalytic pocket of Dha_MEK1, we depicted the dynamic conformational changes of the active segment (Figure 14). Compared with Dha_MEK1, we noticed that in the Dha_MEK1_Sel system, the pocket was almost completely blocked. This closure would suppress the activation of Dha_MEK1 and, therefore, would inhibit downstream ERK activation. Conversely, in the Dha_MEK1_Cob system, the active segment moves downwards, fully exposing the pocket, enhancing the activation of Dha_MEK1. Lastly, the Dha_MEK1_Tra and Dha_MEK1_Bin systems exhibit similar behavior as the Dha_MEK1 system, suggesting that Trametinib and Binimetinib may not affect Dha_MEK1 significantly. In summary, the binding of Selumetinib shifts the catalytic pocket of Dha_MEK1 from an open to a closed state, indicating that it could inhibit Dha_MEK1’s abnormal catalytic function.

The hydrogen bonding interactions between Selumetinib and Dha_MEK1 further confirm the blocking impact of Selumetinib. Table 2 lists the hydrogen bond data for the four protein–ligand complexes (only frequencies exceeding 10% are shown). Analysis of the data reveals that the Dha_MEK1_Sel system exhibits a higher number and probability of hydrogen bonds between the protein and ligand compared to the other three complexes. Specifically, Selumetinib has robust hydrogen bonding interactions with critical residues in the active pocket, including Thr226, Arg227, Phe209, and Asp190. Notably, Asp190 emerges as a key residue with a high hydrogen bond probability in the Dha_MEK1_Sel, Dha_MEK1_Cob, and Dha_MEK1_Bin binding systems.

## 3. Materials and Methods

**Figure 15:**
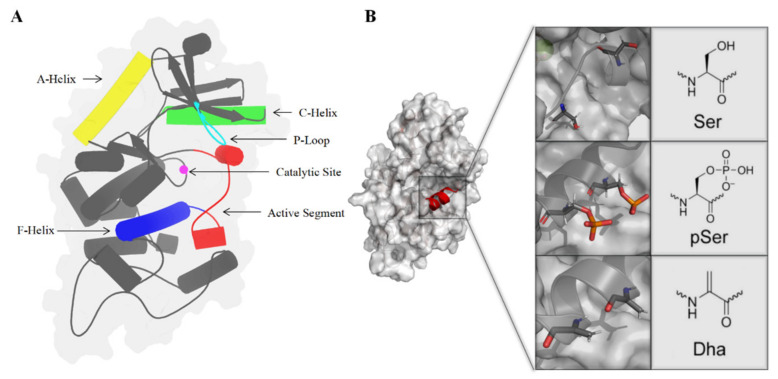
(**A**) Schematic representation of MEK1 structure. (**B**) Structure of MEK1 with active segment highlighted (wild-type, phosphorylated and Dha-modified Ser218 and Ser222 are shown, respectively).

The crystal structure of MEK1 (PDB ID: 3EQD) was obtained from the Protein Data Bank (www.rcsb.org) (Figure 15A). The WT_MEK1 structure was generated with ATP and Mg2+ ligands. Using the WT_MEK1 structure as a template, the phosphorylated MEK1 (PP_MEK1) structure was constructed with phosphorylated serine residues at positions 218 and 222, employing the online CHARMM-GUI (https://charmm-gui.org/, Version 3.7, accessed on 30 December 2021) [42,43]. The Dha-modified MEK1 (Dha_MEK1) structure was generated using Discovery Studio 4.0, where Dha mutations were introduced at the phosphorylated serine residues at positions 218 and 222 (Figure 15B), followed by local Dha optimization. The Dha parameters utilized were based on the CHARMM36 force field parameters reported in a 2014 study [44].

The molecular dynamics simulations were performed using the GROMACS 2021 software package (https://www.gromacs.org/, accessed on 30 January 2021). Three systems, WT_MEK1, PP_MEK1, and Dha_MEK1, were simulated for 1 microsecond each using conventional molecular dynamics simulations. The systems were parameterized with the CHARMM36 force field [45] and were placed in a cubic box with periodic boundaries of 10 × 10 × 10 nm^3^. TIP3P water model was used as the solvent, and 0.15 M NaCl was added to mimic the salt environment in the human body, maintaining the system’s charge neutrality. The energy minimization was carried out for 50,000 steps using the steepest descent method t. Subsequently, 100 ps of NVT (Berendsen temperature coupling with constant particle number, volume, and temperature) and 1000 ps of NPT (Parrinello–Rahman pressure coupling with constant particle number, pressure, and temperature) were performed to equilibrate the system (310 K, 1 bar). The coupling constants for temperature and pressure were set to 0.1 and 2.0 ps, respectively. The long-range electrostatic interactions were described using the Particle Mesh Ewald algorithm with an interpolation order of 4 and a grid spacing of 1.6 Å. The van der Waals interactions were calculated with a cutoff of 12 Å. All bond lengths were constrained using the LINCS algorithm. After initial equilibration, the molecular systems were simulated for 1000 ns with a time step of 2 fs, and the coordinates of all models were saved every 2 ps.

Well-tempered metadynamics (WTMetaD) simulations [46,47] were carried out using GROMACS 2021 and the PLUMED v2.8.0 plugin [48]. Collective variables (CVs) based on the centers of mass (COMs) of three domains were employed, with CV1 measuring the distance between the active segment and the C-helix, and CV2 measuring the distance between the active segment and the G×G××G domain. A Gaussian bias potential filled the potential energy surface with a height and width of 0.1 kcal/mol and 0.1 Å, respectively. Temperature was set to 310 K, and the bias factor was set to 15 for convergence of free energy curves. Gaussian bias potential was added every 2 ps, with one peak per picosecond. Free energy was calculated every 100 Gaussian hills accumulated. The time step for WTMetaD was 2 fs, and simulations ran for 200 ns for the three systems.

We used WTMetaD simulations to sample multiple conformations of Dha_MEK1. Then, clustering analysis based on CV1 was performed, and the most probable structure from the largest cluster was selected as the representative Dha_MEK1 conformation. This representative conformation was subjected to energy minimization. The resulting representative conformation was then used for docking with four FDA-approved inhibitors: Trametinib, Cobimetinib, Binimetinib, and Selumetinib. Crystal structures of the inhibitors were obtained from the Protein Data Bank (Trametinib PDB ID: 7JUR [14], Cobimetinib PDB ID: 7JUS [14], Binimetinib PDB ID: 7M0U [49] and Selumetinib PDB ID: 7JUT [50]). AutoDock 4.2 was used for molecular docking, employing the Lamarckian genetic algorithm. A grid map with dimensions of 48 Å × 48 Å × 48 Å and grid spacing of 0.375 Å was used. Each docking was repeated 10 times to generate multiple conformations. The lowest-energy conformation for each complex was selected and labeled accordingly.

## 4. Conclusions

In the human body, approximately 40% of cancers involve the MAPK signaling pathway, which includes the RAF/MEK/ERK kinase cascade. MEK1, as an intermediate kinase, is a regulatory bottleneck of the MAPK pathway, and its abnormal activation is associated with the development of various cancers. Typically, the abnormal activation of MEK1 is accompanied by mutations or deletions in specific residues. A recent study [19] revealed that the Dha modification can also lead to abnormal activation of MEK1, suggesting its potential carcinogenicity. In recent years, there have been more and more computational studies on MEK1. Notably, researchers have proposed computational models of phosphorylated MEK1 [4] and simulations on its mutation-induced regulation mechanisms [37]. These studies have deepened our understanding of the relationship between the conformation and function of MEK1.

In this study, we first elucidated the mechanism by which the Dha modification causes the abnormal activation of MEK1. Specifically, visual trajectory analysis revealed that the Dha modification opens the catalytic pocket of MEK1, exposing the catalytic residue Asp190. Through RMSF, hedgehog plot, and secondary structure analysis, we found that the Dha modification induces significant movements and conformational changes in the C-helix, Pro-rich region, and active segment. Distance analysis of the three “gatekeeper” segments covering the catalytic pocket and quantitative calculation of the catalytic pocket volume confirmed the opening of the MEK1 catalytic pocket. The structural changes were accompanied by altered interactions between internal residues. Hydrogen bond analysis showed that in the Dha-modified MEK1, there was increased hydrogen bond interaction between the active segment and the C-terminal, with important contributions from Glu114, Glu233, Tyr240, and Arg305. In summary, after being modified by Dha, MEK1 exhibits stable interaction between the active segment and the protein C-terminus, resulting in an open catalytic pocket and an exposed active site and, therefore, a sustained abnormal activation conformation of MEK1. This sustained activated conformation may significantly affect the role of MEK1 in the MAPK signaling pathway. The conformational changes in MEK1 caused by the Dha modification are a kind of allosteric regulation, influencing MEK1’s activity. Through this allosteric effect, MEK1 keeps the continuously open conformation and, therefore, active downstream ERK kinases with a greater tendency. Additionally, the Dha modification is difficult to eliminate, causing false activation of the MAPK pathway and then the dysregulation of cellular functions. In various cancers, most MAPK pathway dysregulation events are caused by MEK1 mutations [51,52].

Subsequently, we investigated the binding of four FDA-approved MEK1 inhibitors for Dha-modified MEK1 and found that only Selumetinib exhibits obvious blocking effects on the catalytic pocket of Dha-modified MEK1. Small-molecule inhibitors can stabilize different conformational states of kinases through specific interactions, thereby achieving the regulation of kinase activity. A recent study explored the effect of Trametinib on the activation of MEK1 with active or inactive mutations, showing that the inhibitor binds weaker with active mutants (P124S and E203K). The allosteric channel in active MEK1 mutants is wider and shorter, leading to easier dissociation from the active mutants [30]. Similarly, RMSF and secondary structure analysis indicated that Selumetinib directly disrupts the secondary structure of the active segment, converting it from a helix to a random coil structure, thereby blocking the active site and closing the catalytic pocket. In contrast, Cobimetinib, Trametinib, and Binimetinib show no significant impact on the catalytic pocket, which may be a potential reason for the resistance of these FDA-approved inhibitors. Analysis of protein–inhibitor interactions revealed that Selumetinib has more binding sites and stronger binding stability with Dha-modified MEK1 protein. Among them, the hydrogen bond interactions of residues in the active pocket, such as Thr226, Arg227, Phe209, and Asp190, contribute the most to the binding between Selumetinib and MEK1.

In a word, this work elucidates the mechanism of Dha modification-induced abnormal activation of MEK1 and predicts the inhibitory effects of FDA-approved MEK1-targeting inhibitors. The findings will contribute to the understanding of the mechanism underlying Dha modification-induced abnormal activation of MEK1 and provide insight for the development of corresponding inhibitors.

## Figures and Tables

**Table 1:** List of hydrogen bond occupancy.

Hydrogen Bond		WT_MEK1	PP_MEK1	Dha_MEK1
Donor	Acceptor	Occupancy	Occupancy	Occupancy
ARG108-Side-NH1	ASP136-Main-O	0.25%	24.25%	13.75%
ARG189-Side-NH2	SER241-Main-O	0.00%	36.00%	40.75%
ARG189-Side-NH2	ASP245-Side-OD1	0.00%	27.00%	26.25%
GLY213-Main-N	TYR240-Side-OH	0.00%	15.00%	48.50%
ARG234-Side-NH2	ASP217-Side-OD2	0.00%	39.50%	64.75%
ARG234-Side-NE	ASP217-Side-OD1	0.00%	44.75%	73.50%
SER244-Side-OG	GLU233-Side-OE2	13.25%	23.00%	76.50%
ARG305-Side-NE	SER228-Side-OG	0.00%	0.00%	26.00%

**Table 2:** List of hydrogen bond occupancy between inhibitors and Dha_MEK1 in the Dha_MEK1_Tra, Dha_MEK1_Sel, Dha_MEK1_Cob, and Dha_MEK1_Bin (only occupancy >10% listed).

Systems	Donor	Receptor	Occupancy
Dha_MEK1_Tra	GLU102-Side-OE2	TRA385-Side-O33	27.99%
	GLU102-Side-OE1	TRA385-Side-O33	21.66%
Dha_MEK1_Sel	ASP190-Side-OD1	SEL385-Side-O3	36.98%
	MET187-Main-O	SEL385-Side-O3	33.33%
	ASP190-Side-OD2	SEL385-Side-O3	22.99%
	PHE209-Main-O	SEL385-Side-O3	14.33%
	MET219-Main-N	SEL385-Side-N4	11.33%
	THR226-Side-OG1	SEL 385-Side-N4	10.33%
	THR226-Main-N	SEL 385-Side-F1	20.33%
	ARG227-Main-N	SEL 385-Side-O3	20.33%
Dha_MEK1_Cob	PHE209-Main-N	COB385-Side-F07	25.61%
	ASP190-Side-OD2	SEL385-Side-LP1	12.13%
	ILE186-Side-CB	COB385-Side-LP1	26.33%
	LYS185-Side-CB	COB385-Side-LP1	21.33%
	GLU114-Side-CG	COB385-Side-LP1	11.33%
Dha_MEK1_Bin	ASP217-Side-OD2	BIN385-Side-O27	19.93%
	ASP217-Side-OD1	BIN385-Side-O27	16.61%
	ILE216-Side-CG1	BIN385-Side-LP1	12.33%
	ASP190-Side-OD1	BIN385-Side-O27	31.00%
	ILE216-Side-CG1	BIN385-Side-LP1	12.33%
	VAL211-Side-CG2	BIN385-Side-LP1	11.00%

## Data Availability

Data is contained within the article.
